# Myocardial dysfunction in the periinfarct and remote regions following anterior infarction in rats quantified by 2D radial strain echocardiography: An observational cohort study

**DOI:** 10.1186/1476-7120-6-17

**Published:** 2008-04-29

**Authors:** Raymond Q Migrino, Xiaoguang Zhu, Mineshkumar Morker, Tejas Brahmbhatt, Megan Bright, Ming Zhao

**Affiliations:** 1 Department of Medicine (Cardiovascular Division), Medical College of Wisconsin, Milwaukee WI, USA; 2 Department of Biophysics, Medical College of Wisconsin, Milwaukee WI, USA; 3 School of Medicine, Medical College of Wisconsin, Milwaukee WI, USA

## Abstract

**Background:**

Heart failure from adverse ventricular remodeling follows myocardial infarction, but the contribution of periinfarct and remote myocardium to the development of cardiomyopathy remains poorly defined. 2D strain echocardiography (2DSE) is a novel and sensitive tool to measure regional myocardial mechanics. The aim is to quantify radial strain in infarcted (I), periinfarct (PI) and remote (R) myocardial regions acutely and chronically following anterior infarction in rats.

**Methods:**

The left anterior coronary artery of male Sprague-Dawley rats (270–370 g) were occluded for 20–30 minutes and 2DSE was performed in the acute setting (n = 10; baseline and 60 minutes post-reperfusion) and in the chronic setting (n = 14; baseline, 1, 3 and 6 weeks). Using software, radial strain was measured in the mid-ventricle in short axis view. The ventricle was divided into 3 regions: I (anteroseptum, anterior and anterolateral), PI – (inferoseptum and inferolateral) and R – (inferior). Infarct size was measured using triphenyl tetrazolium chloride in the acute group.

**Results:**

Following infarct, adverse remodeling occurred with progressive increase in left ventricular size, mass and reduced fractional shortening within 6 weeks. Radial strain decreased not only in the infarct but also in the periinfarct and remote regions acutely and chronically (I, PI, R, change vs. baseline, 60 minutes -32.7 ± 8.7, -17.4 ± 9.4, -13.5 ± 11.6%; 6 weeks -24.4 ± 8.2, -17.7 ± 8.3, -15.2 ± 8.4% respectively, all p < 0.05). Reduced radial strain in periinfarct and remote regions occurred despite minimal or absent necrosis (area of necrosis I, PI, R: 48.8 ± 23, 5.1 ± 6.6, 0 ± 0%, p < 0.001 vs. I).

**Conclusion:**

Following left anterior coronary occlusion, radial strain decreased at 60 minutes and up to 6 weeks in the periinfarct and remote regions, similar to the reduction in the infarct region. This demonstrates early and chronic myopathic process in periinfarct and remote regions following myocardial infarction that may be an under recognized but important contributor to adverse left ventricular remodeling and progression to ischemic cardiomyopathy.

## Background

Left ventricular remodeling from myocardial infarction is a leading cause of heart failure and death in the United States [1-3]. Following myocardial infarction, adverse remodeling occurs acutely within hours [4], continues weeks to months following the event [5,6] and is the most significant predictor of survival. Ischemic cardiomyopathy is a specific cardiomyopathy defined by the World Health Organization as a dilated cardiomyopathy with impaired contractile performance not explained by the extent of coronary artery disease or ischemic damage [7]. The mechanical dysfunction underlying the development of ischemic cardiomyopathy remains poorly understood. Prior studies have demonstrated reduced contractility in normally perfused myocardial regions adjacent to the infarct with preserved function in remote regions [8-10]. The expansion of this periinfarct zone is believed to cause increased dynamic wall stress and leads to adverse remodeling [9]. This myopathic process in normally perfused myocardium was reported to be localized initially to the myocardium abutting the infarct but later extends during the remodeling process to contiguous segments making these hypocontractile [3,8,11]. It has recently been shown that myocardial strain measured by 2-dimensional strain echocardiography (2DSE) is more sensitive in detecting regional myocardial dysfunction compared with conventional means of functional assessment such as thickening or fractional shortening in both ischemic [12] and doxorubicin-induced cardiomyopathy animal models [13]. The 2DSE method relies on motion estimation based on tracking of unique ultrasound speckle patterns within the tissue to calculate myocardial strain [14-16]. 2D strain echocardiography, because it measures the deformation of a myocardial segment, is a more direct measure of myocardial mechanics compared to conventional measures of ventricular function such as ejection fraction/fractional shortening (the latter measure change in cavity size and therefore are indirect measures of ventricular function). The aim of this study is to utilize this sensitive technique to quantify the change and to determine the temporal course of myocardial radial strain following occlusion of the left anterior coronary artery in infarcted, periinfarct and remote regions in rats.

## Methods

### Animal model

**Figure 1:**
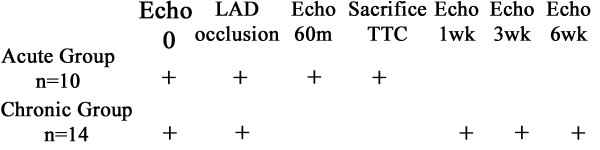
Experimental design.

The trial design is an observational cohort study. Male Sprague-Dawley rats (270–370 g) were used, 10 for the acute and 14 for the chronic experiment (Figure 1). The rats were anesthetized with sodium pentobarbital (40 mg/kg intraperitoneal). The rats underwent tracheotomy, intubated and placed on a rodent ventilator (Hugo Sachs Elektronik-Harvard Apparatus, March-Hugstetten, Germany) as previously published [12]. Left thoracotomy was performed to expose the heart and the proximal left anterior coronary artery was occluded by a suture for 20–30 minutes after which the occlusion was removed to achieve reperfusion. For the acute group, echocardiography was performed at baseline and at 60 minutes following reperfusion. The animals in this group were sacrificed at 60 minutes post-reperfusion and the hearts were excised. 0.5% solution of triphenyl tetrazolium chloride (TTC) was injected retrograde into the aorta and coronary arteries and the heart was fixed in 10% neutral buffered formaldehyde. For the chronic group, the chest incision was closed. For this group, echocardiography was performed at baseline, and 1, 3 and 6 weeks following coronary occlusion. The protocol was approved by the local institutional animal care and use committee.

### Echocardiography

An echocardiograph (Vivid 7, General Electric, Waukesha WI) was used with 11 MHz M12L linear array transducer. The parasternal short axis view of the mid left ventricle was used for the study because of the easy reproducibility of this view in this species for serial studies owing to well-defined anatomic landmarks (prominent papillary muscles and the anterior and posterior insertion of the right ventricle). Image depth was 2–2.5 cm with 234–256 frames per second acquisition using second harmonic imaging. Electrocardiographic gating was used. The images were sent to a workstation with software (EchoPAC workstation with Q analysis software, General Electric, Waukesha WI) for analyses.

#### Radial strain

The left ventricle was divided into 6 segments as defined by the American Society of Echocardiography [17]. The method of analysis has been previously described [12]. For each cardiac cycle (defined from peak of the R wave to the following R wave), the endocardial border was traced at end-systole. The outer border was adjusted to approximate the epicardial contour. The software automatically selected acoustic objects within the myocardium for tracking and computed radial strain and radial strain rate in 6 segments of the mid left ventricle throughout the cardiac cycle. Endsystolic radial strain and peak early diastolic radial strain rate were obtained for each segment. End systole was defined as the point when the radial strain rate becomes zero from a positive value. Three consecutive heart beats were measured and the average obtained. From TTC data demonstrating that the model produced infarction in the anteroseptal, anterior and anterolateral segments, the heart was divided into 3 regions for analyses: infarct region (anteroseptal, anterior and anterolateral segments), periinfarct region (inferolateral and inferoseptal segments) and remote region (inferior segments).

#### Measurement of left ventricular remodeling

From the 2D image of the mid left ventricle, anatomical M-mode through the plane of the anteroseptum and inferolateral segments was used to measure left ventricular internal diameter at diastole (LVIDd) and systole (LVIDs). Fractional shortening (FS) was calculated using the formula: FS = (LVIDd-LVIDs)/LVIDd × 100%. Left ventricular mass was derived from the anteroseptal thickness (AST) and inferolateral thickness (ILT) using the formula: 0.8 [1.04{(ILWT+LVID+AST)^3^-LVID^3^}]+0.6 [18]. Three consecutive heart beats were measured and averaged.

### Histology

Following TTC injection, the heart was sectioned in 1 mm thick short axis slices similar in orientation as the echocardiography slices. The midventricular section with corresponding papillary muscle as landmark was chosen. The slice was divided radially into 6 equal segments with the orientation adjusted to correspond to the echocardiographic images using the papillary muscles and the insertion points (anterior and posterior) of the right ventricle to the left ventricle as markers. Pale areas (lack of TTC stain) corresponding to areas of infarct were measured using software (ImageJ, National Institutes of Health, Bethesda MD).

### Data and statistical analyses

Data are expressed as means ± standard deviation. Continuous variables measured serially were compared using 2 way repeated measures analysis of variance followed by pairwise multiple comparison procedures (Holm-Sidak method) (Sigmastat 3.5, Systat Software Inc., San Jose CA). Pearson's correlation was used to describe the relationship between radial strain in the infarcted and periinfarct/remote regions. A significant p value was set at 0.05.

## Results

### Left ventricular remodeling

**Figure 2:**
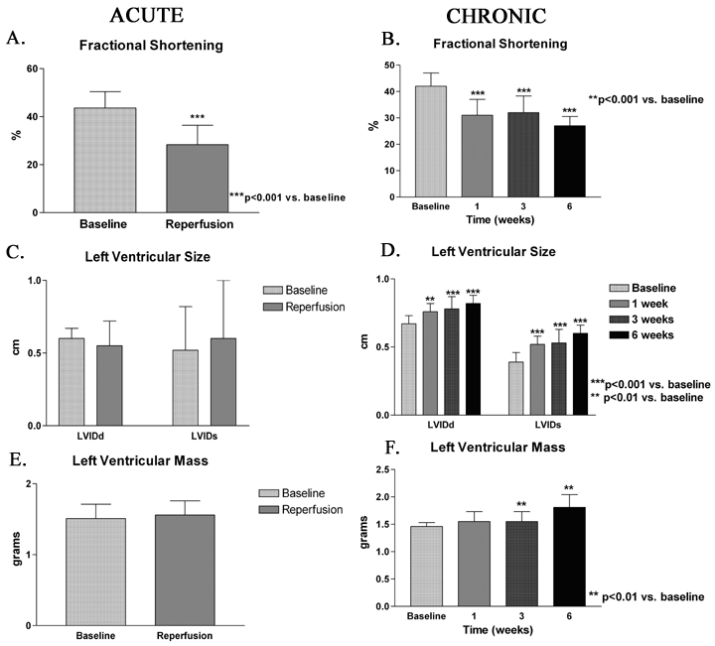
Left ventricular remodeling in the acute (A, C, E) and chronic group (B, D, F). A and B. There is significant reduction in fractional shortening following ischemia-reperfusion and persists up to 6 weeks. C and D. There is a trend towards increased end systolic dimension 60 minutes following ischemia. There is progressive dilation of the left ventricle from 1–6 weeks. E and F. The estimated left ventricular mass increased at 3 weeks and progressed at 6 weeks. (LVIDd-left ventricular internal diameter in diastole, LVIDs-left ventricular internal diameter in systole).

Following reperfusion, there was no significant change in left ventricular diastolic dimension but there was a trend towards increased end-systolic diameter (Figure 2). As a result, fractional shortening was significantly reduced following ischemia-reperfusion. Left ventricular size (diastolic and systolic dimensions) progressively increased from 1 to 6 weeks. There was progressive decrease in fractional shortening and increase in left ventricular mass from 1 to 6 weeks.

### Infarct size

**Figure 3:**
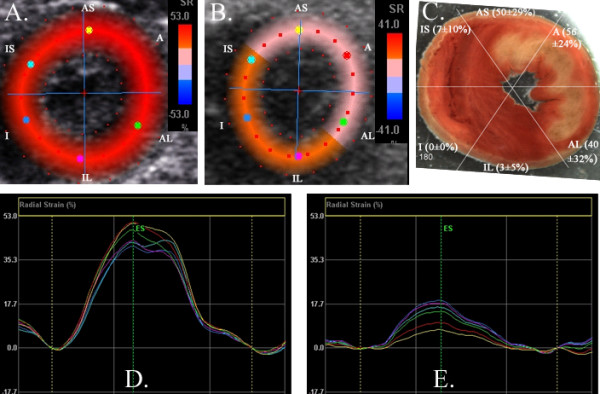
Radial strain and histology. A and B. Parametric image representation of radial strain at mid left ventricle at baseline (A) and following 30 minutes of ischemia/60 minutes of reperfusion (B). The color represents radial strain at endsystole with scale to the right of the image. There is reduction in radial strain in all segments but greatest in the anteroseptum, anterior and anterolateral segments. D and E. Graphical representation of radial strain by time in the cardiac cycle at baseline (D) and following ischemia-reperfusion (E) demonstrates reduction in systolic strain in all segments. C. TTC histology at mid ventricle. There are pale areas in the anteroseptal (AS), anterior (A) and anterolateral (AL) segments corresponding to lack of TTC and myocardial necrosis. The inferolateral (IL), inferoseptal (IS) and inferior segments show no necrosis. The numbers in each region represent the mean ± standard deviation of the area of necrosis in each segment.

Occlusion of the left anterior coronary artery produced necrosis in the anteroseptal, anterior and to a lesser degree in the anterolateral region (Figure 3). There was minimal necrosis in the inferolateral and inferoseptal regions. There was no necrosis in the inferior region. The areas of necrosis were 48.8 ± 23, 5.1 ± 6.6 and 0 ± 0% for infarct (anteroseptal, anterior and anterolateral segments), periinfarct (inferolateral and inferoseptal segments) and remote (inferior) regions, respectively (both p < 0.001 versus infarcted region).

### Radial strain

**Figure 4:**
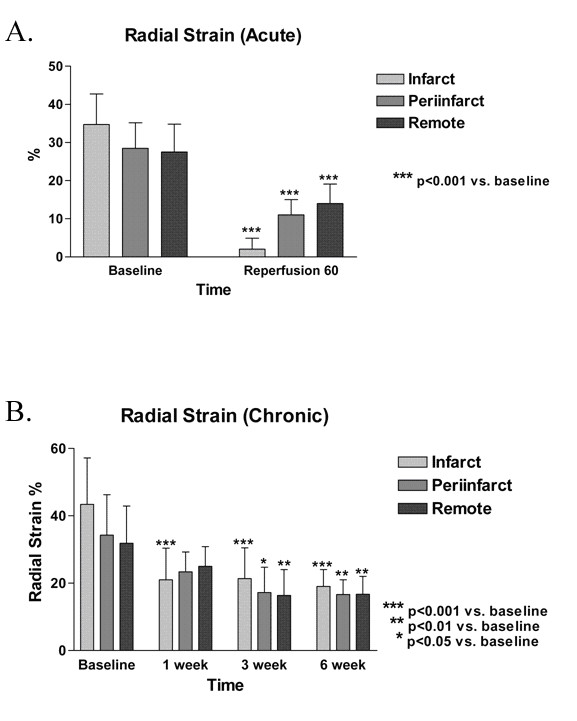
Radial strain. A. Acute group. There is significantly reduced radial strain compared to baseline in the infarct, periinfarct and remote regions. There is no significant difference in radial strain following ischemia-reperfusion among the three regions. B. Chronic group. There is reduced radial strain from baseline in the infarct region starting at 1 week. Reduced radial strain is seen at 3 and 6 weeks compared to baseline in the periinfarct and remote regions. No significant difference in radial strain is seen among the three groups at 1, 3 and 6 weeks.

**Figure 5:**
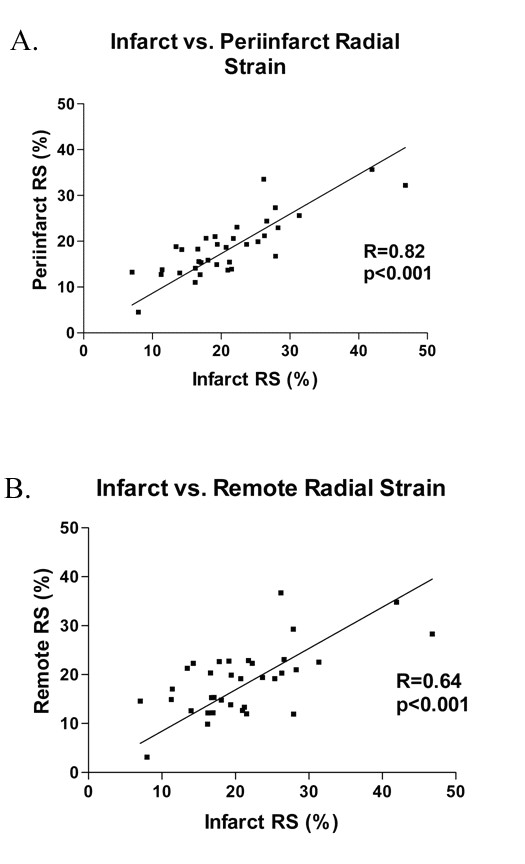
Correlation between infarct region and periinfarct region radial strain (A) and remote region (B) in the chronic group. There is significant correlation between radial strain in the infarct region and those in the periinfarct and remote regions.

Based on the TTC findings, the left ventricle was divided into infarct, periinfarct and remote regions. In the acute group, there was significant reduction in radial strain 60 minutes following ischemic injury in all 3 regions (Figure 4). The changes in radial strain from baseline were -32.7 ± 8.7, -17.4 ± 9.4, -13.5 ± 11.6% for infarct, periinfarct and remote regions, respectively (all p < 0.001 versus baseline). In the chronic group, the reduction in radial strain persisted in all 3 regions up to 6 weeks, with comparable reduction in all 3 regions. The changes in radial strain at 6 weeks from baseline were -24.4 ± 8.2, -17.7 ± 8.3, -15.2 ± 8.4% for infarct, periinfarct and remote regions, respectively (all p < 0.01 versus baseline). There is significant correlation in radial strain between infarcted versus periinfarct or remote regions (Figure 5).

**Figure 6:**
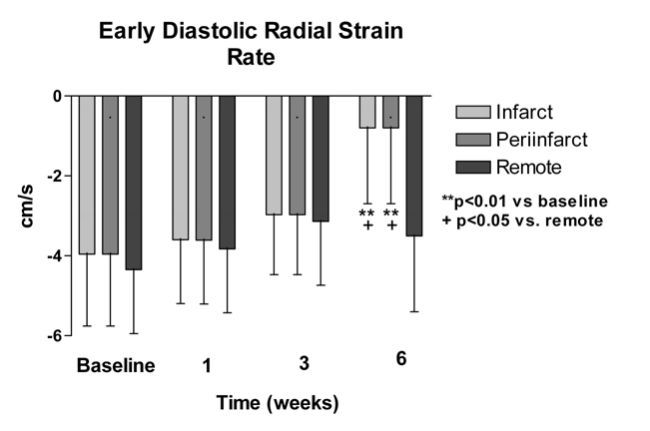
Early diastolic peak strain rate in the chronic group. There is progressive reduction in early diastolic peak strain rate in the infarct and periinfarct zone but not in the remote region, with significant difference from baseline at 6 weeks post infarction.

As a measure of regional diastolic function, the early diastolic radial strain rate showed progressive reduction with time and was significantly reduced by 6 weeks in the infarct and the periinfarct region compared to baseline and the remote region (Figure 6).

## Discussion

Coronary artery disease and myocardial infarction remain the leading causes of mortality in the United States [1]. Adverse left ventricular remodeling occurs within hours following infarction and is one of the most important prognosticators for short and long term survival [4]. Heart failure is a sequela of progressive cardiac enlargement and dysfunction, processes that continue months to years after the event [5,6]. The mechanical and mechanistic bases underlying the development of ischemic cardiomyopathy, defined officially as dilated cardiomyopathy with impaired contractile performance not explained by extent of coronary artery disease or ischemic damage [7], are not well understood. Investigators showed that adverse remodeling occurs not only because of myocyte loss due to ischemic injury, but also because of a myopathic process in adjacent normally perfused periinfarct border zones [3,8-11]. Beginning at the periphery of the infarct region, with time, there is extension of the hypocontractile but perfused borderzone myocardium as the heart remodels [8]. The mechanisms underlying this surrounding myocardial dysfunction are not well-known. A change in local geometry with reduction in endocardial curvature was postulated to cause increased dynamic wall stress [9] but further 3-dimensional assessment of wall thickness and curvature puts in question whether increased regional wall stress occurs in this region [10]. Matrix metalloproteinases were induced >300% and tissue inhibitors of matrix metalloproteinases were inhibited in infarcted and periinfarct regions pointing to the possible contribution of disrupted extracellular matrix in remodeling [19]. Myocardial stretch leads to oxidant stress and induction of apoptosis [20] and may play a role in the myopathic process.

The current study utilized TTC staining to demarcate regions with ischemic injury from coronary occlusion. This technique is based on the loss of myocardial dehydrogenase activity by necrotic myocyte and has been validated using histology and electron microscopy to reliably quantify recent infarction [21]. The ischemia time used has been shown to cause irreversible damage in the coronary territory perfused, and the infarct area obtained in this study is consistent with prior report that 30 minute ischemia in the same artery resulted in infarction of 54% of region at risk [22]. Based on the TTC data, the investigators are confident that the anatomical delineation between infarct, periinfarct and remote zones as regards coronary perfusion is valid. Because the TTC necrotic areas in the periinfarct and remote zones are minimal to none, the observed abnormality in myocardial mechanics in these regions is unlikely to be ischemic (secondary to reduced coronary perfusion) in nature.

Although the abnormal strain in the periinfarct region is consistent with prior observations [8-10], the current study has novel finding of reduced radial strain in the remote myocardial region both acutely and chronically. Because the abnormality persisted weeks after the ischemic insult, the abnormal radial strain in remote regions cannot be attributed to transient myocardial stunning. Prior investigation in a sheep model demonstrated reduced fractional shortening in the borderzone, but not in the remote region following ischemia, but from 2–8 weeks post infarct, the rate of myocardial expansion and enlargement was not significantly different between infarct, periinfarct and remote regions [8]. The discrepancy may possibly be explained by the enhanced sensitivity of 2DSE in detecting abnormal regional myocardial mechanics. 2D strain echocardiography has recently been shown to be more sensitive in evaluating cardiomyopathy compared to conventional means of functional assessment. In a rat chronic doxorubicin cardiomyopathy model, 2DSE derived radial strain was shown to detect cardiomyopathy 4 weeks earlier than fractional shortening, with detectable changes in radial strain corresponding to the onset of histologic evidence of myocyte apoptosis [13]. There was also significant negative correlation between radial strain and degree of myocardial fibrosis. In the same cohort of rats used in this study, it was shown that 2DSE strain was more sensitive and specific than segmental thickening in predicting area of infarct by TTC [12]. In another cardiomyopathy model, following massive whole body irradiation, rats demonstrated earlier reduction in radial strain as compared to fractional shortening/ejection fraction, and this reduction was associated with histologic evidence of myocardial fibrosis and reduction in coronary microcirculation [23].

Early recognition of cardiomyopathy not only in the periinfarct but also in the remote region may be important in attenuating left ventricular remodeling. Surgical means of restraining ventricular expansion through the use of external cardiac support device have shown promise in an animal model for reducing global remodeling [3]. Pharmacological inhibition of matrix metalloproteinases in animal models have been shown to attenuate remodeling[19,24-27]. Addition of nitric oxide releasing drug C87-3754 reduced oxidant stress and apoptotic injury in overstretched papillary muscles [20]. Greater understanding of the mechanisms behind the cardiomyopathy in the remote and periinfarct regions is needed in order to develop novel therapeutic options.

In contrast to early reduction in systolic radial strain, the peak early diastolic radial strain rate had late reduction at 6 weeks and was only significant in the infarct and periinfarct regions. Impaired early diastolic strain rate suggests diastolic dysfunction with abnormal myocardial relaxation and compliance and has been shown to have the strongest impact on cardiac mortality [28-30]. The finding therefore suggests enhanced regional diastolic dysfunction in infarcted and periinfarct regions compared to remote regions. It remains unclear why diastolic function, but not systolic function, is preserved in the remote region. It is also unclear why regional diastolic function (peak early diastolic strain rate) becomes abnormal in the infarct and periinfarct regions only in the later stages of recovery, unlike the early abnormality seen with regional systolic function (end systolic radial strain).

The rat model used is an established model of ischemic injury [22] and the ventricular remodeling observed is similar to human disease [4-6]. The use of 2D radial strain echocardiography to determine regional myocardial viability has been validated in the same rat model using TTC histology [12]. In human subjects with established infarction, 2D strain echo has similarly been useful in distinguishing regional myocardial viability as defined by gadolinium late enhancement by magnetic resonance imaging [31]. Therefore, the use of this novel technique in defining myocardial dysfunction in periinfarct and remote regions may potentially be applicable to human disease but needs to be formally validated in a clinical study.

## Limitations

Although TTC histology was available for the acute group, it was not available for the chronic group. Because similar operative techniques were utilized between the acute and chronic groups, it is reasonable to assume that the regional designation in the acute also applies to the chronic group. Invasive hemodynamic monitoring was not available to determine the effect of intraventricular pressure on regional wall stress. The study was limited in not being able to determine the mechanistic basis for the reduction in radial strain in the periinfarct and remote regions. The study is also limited in the ability to distinguish the contribution of regional tethering of the akinetic infarcted myocardium in the reduction of radial strain in the periinfarct region (a pseudo-reduction) versus intrinsic myocardial dysfunction in the region (a true reduction). However, several lines of evidence suggest that a true reduction in radial strain is occurring. First, the significant reduction in radial strain in the contralateral remote region is difficult to explain by the passive effects of tethering. Second, progressive dilatation in all regions and not just in the infarcted region cannot be explained by passive effects from infarct region. Third, ischemic injury in the LAD territory that did not result in infarction (therefore there was absence of akinetic infarcted tissue to produce passive tethering) similarly caused reduction in strain in both radial and circumferential directions in the periinfarct and remote regions in a similar rat model of injury [12]. An additional technical point is that unlike Doppler based longitudinal strain rate analysis, speckle-tracking based strain measurement is independent of passive translational motion and regional LV variation [14]. A potential limitation of 2D strain echocardiography is the dependence on good quality B mode images in order for the assumption that natural acoustic markers change position in conjunction with underlying tissue to be true [15]. In this particular study, the endocardial border definition by visual inspection was excellent and the software tracking scores that provide objective evidence of the software's ability to evaluate the acoustic speckles [12,16] were good to excellent providing confidence in the strain measurements.

## Conclusion

The study demonstrates that abnormal radial strain signifying myocardial dysfunction occurs within 1 hour and persists up to 6 weeks in periinfarct and remote myocardial regions following occlusion of the left anterior coronary artery in rats. The degree of myocardial dysfunction in periinfarct and remote regions is closely related to the dysfunction in the infarct region. This is the first study that we know of that demonstrates abnormal function not only in the periinfarct borderzone but also in remote regions, both acutely and chronically. The persistent myocardial dysfunction in the periinfarct and remote regions together with the dysfunction in the infarct region were associated with progression of left ventricular enlargement and adverse remodeling. The mechanism underlying this observation may be important in attenuating adverse remodeling following myocardial infarction and preventing heart failure.

## Competing interests

The authors declare that they have no competing interests.

## Authors' contributions

RQM, XZ, MZ conceptualized the study, carried out the studies, analyzed the data and participated in manuscript preparation. MM, TB and MB participated in data analysis and participated in manuscript preparation. All authors read and approved the final manuscript.
